# Early and Next-Generation KIT/PDGFRA Kinase Inhibitors and the Future of Treatment for Advanced Gastrointestinal Stromal Tumor

**DOI:** 10.3389/fonc.2021.672500

**Published:** 2021-07-12

**Authors:** Sebastian Bauer, Suzanne George, Margaret von Mehren, Michael C. Heinrich

**Affiliations:** ^1^ Department of Medical Oncology, West German Cancer Center, Essen University Hospital, University of Duisburg-Essen, Essen, Germany; ^2^ Department of Medical Oncology, Dana-Farber Cancer Institute, Boston, MA, United States; ^3^ Department of Hematology and Medical Oncology, Fox Chase Cancer Center, Philadelphia, PA, United States; ^4^ Department of Medicine, Portland VA Health Care System and OHSU Knight Cancer Institute, Oregon Health and Science University, Portland, OR, United States

**Keywords:** gastrointestinal stromal tumor (GIST), KIT, PDGFRA, ripretinib, avapritinib

## Abstract

The majority of gastrointestinal stromal tumors (GIST) harbor an activating mutation in either the KIT or PDGFRA receptor tyrosine kinases. Approval of imatinib, a KIT/PDGFRA tyrosine kinase inhibitor (TKI), meaningfully improved the treatment of advanced GIST. Other TKIs subsequently gained approval: sunitinib as a second-line therapy and regorafenib as a third-line therapy. However, resistance to each agent occurs in almost all patients over time, typically due to secondary kinase mutations. A major limitation of these 3 approved therapies is that they target the inactive conformation of KIT/PDGFRA; thus, their efficacy is blunted against secondary mutations in the kinase activation loop. Neither sunitinib nor regorafenib inhibit the full spectrum of KIT resistance mutations, and resistance is further complicated by extensive clonal heterogeneity, even within single patients. To combat these limitations, next-generation TKIs were developed and clinically tested, leading to 2 new USA FDA drug approvals in 2020. Ripretinib, a broad-spectrum KIT/PDGFRA inhibitor, was recently approved for the treatment of adult patients with advanced GIST who have received prior treatment with 3 or more kinase inhibitors, including imatinib. Avapritinib, a type I kinase inhibitor that targets active conformation, was approved for the treatment of adults with unresectable or metastatic GIST harboring a PDGFRA exon 18 mutation, including PDGFRA D842V mutations. In this review, we will discuss how resistance mutations have driven the need for newer treatment options for GIST and compare the original GIST TKIs with the next-generation KIT/PDGFRA kinase inhibitors, ripretinib and avapritinib, with a focus on their mechanisms of action.

## Introduction

Gastrointestinal stromal tumors (GISTs) arise from the interstitial cells of Cajal (1, 2); they occur primarily within the stomach (~56%) and small intestine (~32%) but can arise anywhere in the gastrointestinal (GI) tract (3). Although rare, GISTs are the most common sarcoma of the GI tract (3), with reported incidences between 10 and 15 cases per million annually (3). GISTs are best categorized by molecular subtype, which have differing clinical characteristics and treatment response (4). The majority of GISTs harbor activating mutations in 1 of 2 receptor tyrosine kinases (RTKs): KIT (approximately 69%–83%) (4),or platelet-derived growth factor receptor α (PDGFRA; approximately 5%–10%) (5, 6). The 10%–15% of GISTs without *KIT/PDGFRA* mutations are a heterogeneous group, historically referred to as “wild-type”, (4, 7) before disease-defining genomic events were identified. “Wild-type” or preferably non-*KIT/PDGFRA*-mutant GISTs may have a succinate dehydrogenase complex deficiency (8), or harbor other mutations, such as activating mutations of *BRAF* or loss-of-function of *NF1*, that lead to activation of the PI3K/mTOR and/or the RAS/RAF/MAPK pathways (7, 9, 10).

The identification of RTK mutations in GIST led to the use of the tyrosine kinase inhibitors (TKI) for the treatment of advanced GIST (11). While the use of TKIs significantly improves progression-free survival (PFS) and overall survival (OS) in patients with advanced GIST (10), the inevitable development of TKI resistance remains an ongoing challenge (12). Recently, next-generation TKIs with novel mechanisms of action (MOA) have been developed to specifically address these challenges (13, 14). In this review, we will discuss how specific classes of mutations have driven the need for newer treatments for GIST and compare historical and next-generation KIT/PDGFRA kinase inhibitors with a focus on their MOA.

## GIST Is Commonly a KIT- or PDGFRA-Oncogene Driven Disease

Most GISTs are driven by oncogenic *KIT*- or *PDGFRA*-activating mutations (10). Both KIT and PDGFRA are members of the class III tyrosine kinase family (15). Gain of function mutations in either the KIT or PDGFRA receptor leads to constitutive, ligand-independent activation, which alters cell proliferation, differentiation, apoptosis, and survival by regulating downstream signaling pathways (4).

### KIT Mutations

Expression of KIT is important for cellular survival and proliferation, particularly in hematopoietic cells, melanocytes, mast cells, and interstitial cells of Cajal (11, 16). The *KIT* proto-oncogene maps to 4q12-13 and encodes a 145-kDA transmembrane RTK, KIT (aka CD117) (11, 17). KIT is activated through binding of its cognate ligand, stem cell factor, to its extracellular domain, inducing receptor homo-dimerization and activation of the intracellular kinase domain (18). Kinase activation initializes downstream signaling pathways, such as the JAK–STAT3, PI3K–Akt–mTOR, and RAS–MAPK pathways, which are important in regulating cellular functions such as cell proliferation, differentiation, and apoptosis (4). Gain of function mutations in *KIT* are a key oncogenic driver in approximately 80% of GISTs (4), and result in ligand-independent kinase activation (4). Primary mutations in *KIT* affect exons encoding the functional domains of the RTK (exons 8, 9, 11, 13, 17) (11). The majority of mutations occur in exon 11 (70%–80%), which encodes the juxtamembrane domain, leading to disruption in auto-inhibitory function and resulting in increased auto-activation of the kinase (17, 19). Approximately 10% of mutations occur in exon 9, which encodes a portion of the extracellular domain, and mostly consist of a typical duplication mutation of codons 502 and 503 (20). Primary mutations are also found in exon 13 (which encodes the ATP-binding region) and exon 17 (which encodes the activation loop), with an occurrence of about 1% each and less frequently in exon 8, encoding part of the extracellular domain (4, 17, 19).

### PDGFRA Mutations

*PDGFRA* is the second most commonly mutated oncogene in GIST. PDGFRA and KIT are highly homologous, activating similar downstream signal transduction pathways (21). Primary *PDGFRA* mutations occur mainly in exons 18 and 12 and more rarely in exon 14 (6). Exon 18 encodes the activation loop and is the most frequent site for *PDGFRA* mutation (~6%) (5, 6, 22). A single mutation, D842V, is the most common exon 18 mutation and detected in 62.6% of *PDGFRA*-mutated tumors (5, 6). Mutations affecting exon 12 (encoding the juxtamembrane domain) and exon 14 (encoding the ATP-binding domain) are rare, identified in approximately 1%–2% and <0.1% of GISTs, respectively (5, 6, 22).

## Early Targeted Treatments to Inactivate Mutated KIT/PDGFRA

KIT/PDGFRA are logical therapeutic targets as the key oncogenic drivers expressed in the majority (85%–90%) of GIST, especially given the minimal activity of conventional treatments such as chemotherapy or radiation for the treatment of advanced GIST. The first early targeted therapies for advanced GIST include the type II kinase inhibitors (which bind the inactive confirmation) (23) imatinib, sunitinib, and regorafenib, which are approved for first-, second-, and third-line treatment, respectively (24–26).

### Imatinib

Imatinib (formerly STI571) is an oral, small-molecule TKI that was originally developed for the treatment of Philadelphia-chromosome-positive chronic myeloid leukemia (27, 28). Imatinib is a competitive inhibitor of the ATP-binding site of certain RTKs including KIT, PDGFRA, ABL kinase, and the chimeric BCR-ABL fusion oncoprotein of chronic myeloid leukemia (29). When KIT is in the inactive conformation, imatinib can occupy the ATP-binding site and prevent substrate phosphorylation and inhibit downstream signal transduction (29).

Multiple clinical trials demonstrated the efficacy of imatinib in treating advanced GIST, leading to its approval for first-line treatment in 2001 (27). The first report of imatinib efficacy was a case report of imatinib (400 mg daily) resulting in a complete metabolic response in a patient with metastatic GIST who had failed to respond to conventional sarcoma therapies (30). After establishing safety and initial efficacy of imatinib in phase 1 testing in advanced GIST (31), an open-label randomized phase 2 trial randomized patients to receive either imatinib 400 or 600 mg once daily. Overall, 53.7% of patients had a partial response and 27.9% had stable disease (32). Imatinib was generally well tolerated, though most patients experienced mild to moderate adverse events (AEs) (32). Common AEs included edema (74.1%), nausea (52.4%), diarrhea (44.9%), myalgia or musculoskeletal pain (39.5%), fatigue (34.7%), dermatitis or rash (30.6%), headache (25.9%), and abdominal pain (25.9%) (32). Two large phase 3 trials compared treatment with imatinib 400 mg once daily to 400 mg twice daily in patients with unresectable or metastatic GIST. In the imatinib 400 mg daily group of the first trial, 5% had a complete response, 45% had a partial response, and 32% had stable disease. In the higher dose group (400 mg twice daily), 6% had a complete response, 48% had a partial response, and 32% had stable disease (33). A second large phase 3 trial, S0033, showed similar antitumor results in advanced GIST, with a median PFS (mPFS) of 18 months and 20 months and an OS of 55 months and 51 months on imatinib 400 mg once daily and twice daily, respectively (34). These results were dramatically improved compared to cytotoxic chemotherapy for advanced/metastatic GIST, which had minimal responses rates (0%−5%) and very short PFS, indicating the futility of standard sarcoma regimens for the treatment of GIST (32).

Despite the high response rates with front-line imatinib, disease progression still occurs in the majority of patients (35). Disease progression as the initial response to imatinib (i.e., during the first 6 months of treatment) is considered primary resistance, while disease progression that occurs after an initial response or stable disease is considered secondary resistance (11). Imatinib primary resistance was first reported by Demetri et al., who noted that 5% of patients had primary resistance to imatinib within 2 months (32). Overall, approximately 10% of GISTs have primary resistance to imatinib (35), which is correlated with the KIT/PDGFRA mutational status. A few other primary mutations are associated with resistance, such as *PDGFRA* exon 18 RD841-842KI or the primary *KIT* exon 17 N822K mutation (5, 36). Tumors that harbor *PDGFRA* D842V mutations confer unequivocal resistance regardless of imatinib dose (35). Relative primary resistance has also been observed in patients with exon 9 mutations (37); however, a higher dose of imatinib (400 mg twice daily) improves the response rate and PFS of this GIST genotype (38).

In most cases, KIT-mutant GISTs develop secondary resistance to imatinib as a result of the emergence of sub-clones harboring secondary KIT mutations (39). Secondary mutations most commonly occur in KIT exons 13 and 14 (encoding the ATP/drug-binding site) or exons 17 and 18 (encoding the activation loop) (11). Secondary mutations within the ATP-binding domain and activation loop cause resistance by different mechanisms (36). Mutations in the ATP-binding domain are thought to directly inhibit imatinib binding, whereas activation loop mutations are thought to stabilize the kinase in the active formation to which imatinib cannot bind (35). In 31 patients treated with imatinib and undergoing surgical resection, 15 tumors showed secondary resistance (40). Secondary mutations were identified in tumors from 7 (46%) of these patients; the majority had *KIT* exon 17 mutations, but *KIT* exons 13 and 14 mutations were also identified (40). In a separate study, 79 samples from 43 patients with advanced GIST were examined pre- and post-imatinib treatment. Of 33 patients with secondary resistance, 22 (67%) had 1 or more secondary mutations that included *KIT* exon 17 (15 patients), *KIT* exon 13 (7 patients), and exon 14 (1 patient) (41). Many of these samples were from tumor biopsy specimens and likely underestimate the true frequency of secondary mutations in patients with clinical imatinib resistance. In addition, substantial intra- and inter tumor mutational heterogeneity has been noted using sensitive sequencing technologies (12).

### Sunitinib

Sunitinib (formerly SU11248), a small-molecule, multitarget TKI, was approved in 2006 as second-line treatment for advanced GIST after imatinib failure. Originally developed as a treatment for acute myeloid leukemia and advanced renal cell carcinoma, sunitinib is a potent competitive inhibitor of the ATP-binding sites of vascular endothelial growth factor receptor (VEGFR)-1, VEGFR-2, FMS-like tyrosine kinase-3, KIT, and PDGFRs (42–45).

In preclinical *in vivo* models, sunitinib exhibited dose-dependent antitumor activity (42, 44). In patients with advanced GIST who had progressed on imatinib, the safety and efficacy of sunitinib therapy was tested in an open-label, dosing-ranging phase 1/2 trial that enrolled 97 patients (46). The maximum tolerated dose was determined to be 50 mg daily, administered on a 4 weeks on/2 weeks off schedule, after 2 of 4 patients at 75 mg daily experienced dose-limiting toxicities (46). A clear clinical benefit was shown with sunitinib use at follow-up, with 7 (7%) of patients having a partial response and 45 (46%) stable disease (46). Overall, the mPFS was 7.8 months and the median OS was 19 months (46). Sunitinib had an acceptable safety profile with mostly mild to moderate treatment-emergent AEs (TEAEs). In a pivotal double-blind, placebo-controlled randomized phase 3 trial, the safety and efficacy of sunitinib as a second-line treatment in GIST was confirmed (47). Overall, the mPFS for patients on sunitinib was 5.6 months compared with 1.4 months for patients on placebo [hazard ratio (HR) 0.33; 95% confidence interval (CI) 0.24−240.47, p <0.0001] (47). A total of 14 (7%) patients had a partial response, and 120 (58%) had stable disease with sunitinib therapy. In the placebo arm, none had a partial response and 50 (48%) had stable disease (47). Sunitinib showed an acceptable safety profile with most AEs being mild to moderate according to Common Terminology Criteria for Adverse Events. However, low-grade toxicities such as stomatitis and enteral mucositis impact quality of life to a greater degree than the grading would imply as present on a daily basis. In addition, serious treatment-related AEs were reported in 20% of sunitinib-treated patients (47) including hand-foot syndrome, hypertension, and diarrhea as well as hematological AEs (47).

Like imatinib, the efficacy of sunitinib correlates with both primary and secondary mutational status. In a retrospective study of 1124 patients with GIST who progressed on imatinib, those who received sunitinib and had tumors that carried exon 9 *KIT* mutations had significantly longer mPFS (12.3 months) than those with exon 11 mutations (7 months) (48). In the above referenced phase 1/2 trial of sunitinib, primary *KIT* mutations were identified in 83% of tumors (49). Clinical benefit and mPFS were better in patients with GIST exon 9 *KIT* mutations compared with exon 11 mutations (clinical benefit: 58% *vs* 34%; PFS: 19.4 *vs* 5.1 months, p = 0.0005) (49). Most secondary mutations occurred in *KIT* exons 13, 14, and 17 (49). Preclinical studies strongly suggest that sunitinib is highly potent against secondary mutations in exons 13 and 14, while mostly inactive against secondary mutations in exon 17 and 18 (50). Clinical evidence supports these findings in retrospective analyses. Patients with a detectable *KIT* exon 13/14 mutation had significantly longer mPFS compared with patients with a detectable exon 17/18 mutation (49).

### Regorafenib

Regorafenib (formerly BAY 73-4506) is an oral, multitarget TKI that was originally developed for renal cancer with a focus on VEGFR inhibitory function (51). It is a competitive inhibitor of the ATP-binding site for KIT and several other targets, including VEGFR-2, TIE2, PDGFRβ, FGFR, RET, cRAF/RAF1, and BRAF (52). Regorafenib is approved for third-line treatment of advanced GIST after progression on imatinib and sunitinib (25).

In preclinical studies, regorafenib showed potent antitumor activity in multiple *in vivo* cancer models (52). In a phase 2 clinical trial as a third-line or higher in patients with advanced GIST (N = 34), of 33 eligible patients, regorafenib provided a mPFS of 10 months. Overall, 4 (12%) patients had a partial response and 22 (66.7%) had stable disease (53). Regorafenib has a safety profile similar to other TKIs with a comparable target spectrum; the most commonly observed AEs were mostly Grade 1 or 2 and consisted of hand-foot syndrome (85%), fatigue (79%), hypertension (67%), and diarrhea (61%) (53). A placebo-controlled, randomized, phase 3 trial (GRID), confirmed the efficacy of regorafenib as third-line or later treatment in patients with advanced GIST. Regorafenib treatment resulted in a mPFS of 4.8 months compared to 0.9 months on placebo (HR 0.27, 95% CI 0.19–0.39; p <0.0001) (54). In patients on regorafenib, a partial response was observed in 6 (4.5%) and 95 (71.4%) had stable disease *vs* 1 (1.5%) with a partial response and 22 (33.3%) with stable disease in the placebo arm (54). Drug-related AEs of any grade were reported in 98.5% of patients on regorafenib and in 68.2% of patients in the placebo arm (54). Grade 3 or higher AEs were reported in 61.4% of regorafenib-treated patients. Some of these AEs included hypertension (23.5%), hand-foot syndrome (19.7%), and diarrhea (5.3%) (54). Grade 3 events in the placebo arm were less frequent (13.6%): hand-foot syndrome (0), hypertension (3.0%), diarrhea (0) (54).

As with imatinib and sunitinib, tumor mutational status also affects regorafenib efficacy. In a phase 2 trial of patients with *KIT* exon 17 secondary mutations, treatment with third-line regorafenib provided significant clinical benefit, with a mPFS of 22.1 months (55). Using tumor samples obtained from the phase 2 trial and additional cell culture and GIST xenograft studies, the impact of different mutations on tumor response to drug was assessed (56). The results showed regorafenib has a complementary activity profile to sunitinib against secondary *KIT* mutations (56). While regorafenib is effective against many mutations, it has poor efficacy against the *KIT* exon 13 V654A mutation, a common secondary imatinib-resistant mutation. Regorafenib is effective for several exon 17 amino acid substitutions involving residue 816, but is resistant to D816V (56).

**Figure 1:**
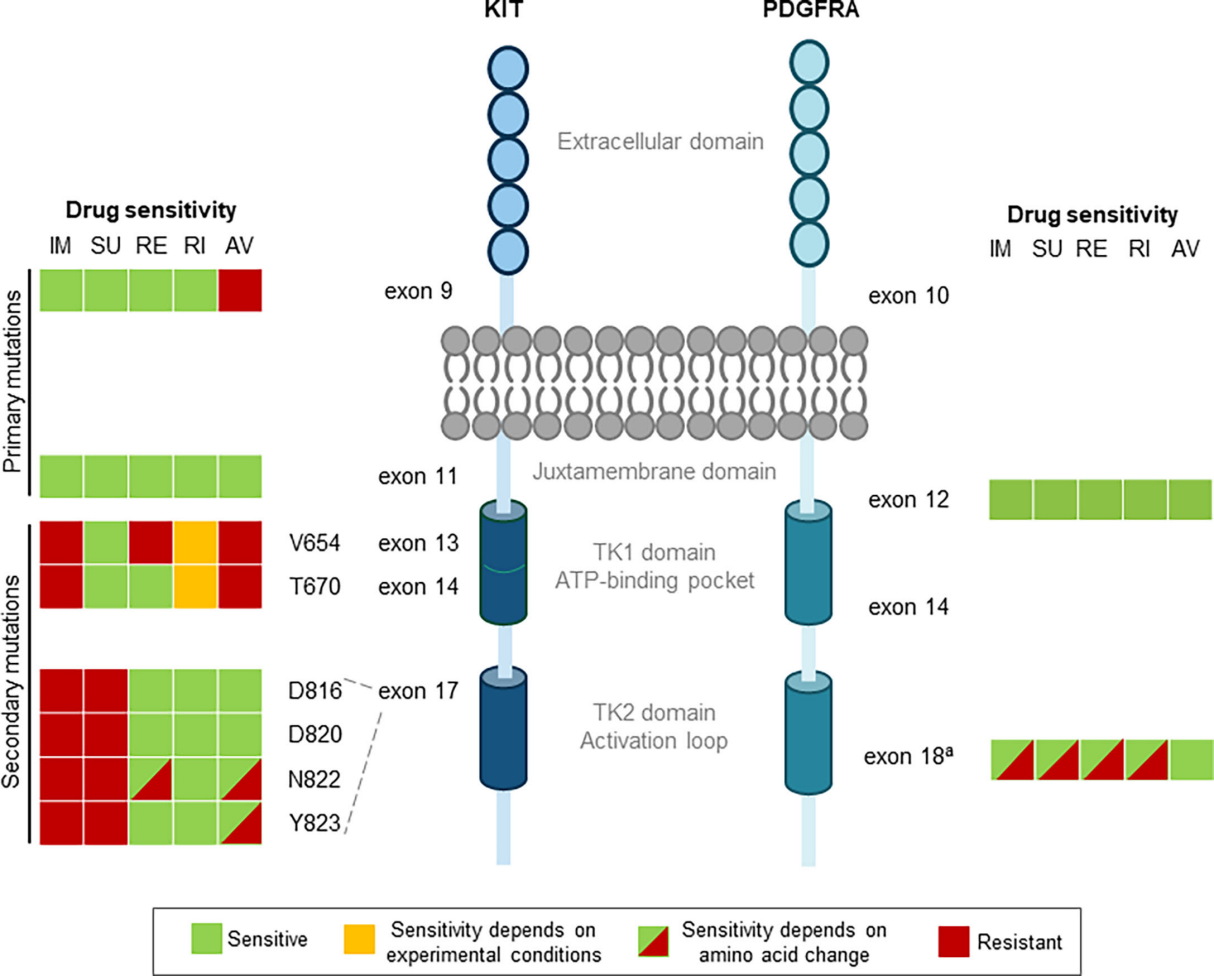
Drug sensitivities for primary and secondary mutations in GIST. Colors denote drug sensitivity: green indicates sensitive, orange indicates sensitivity depends on experimental conditions, red indicates resistant, and red/green hatching indicates that the sensitivity is dependent on the amino acid change. ^a^Approved TKIs are sensitive to non D842V, but only avapritinib and, to some degree, ripretinib are potent against the PDGFRA exon 18 D842V mutation. AV, avapritinib; GIST, gastrointestinal stromal tumors; IM, imatinib; PDGFRA, platelet-derived growth factorreceptor a; RE, regorafenib; RI, ripretinib; SU, sunitinib; TK1, tyrosine kinase domain 1; TK2, tyrosine kinase domain 2.

While these 3 early TKIs have improved the treatment of advanced GIST, treatment resistance remains a challenge as these drugs are not effective against all relevant GIST-associated mutations (**Figure 1**). Two issues are evident. First, all 3 TKIs are type II multikinase inhibitors, which bind to the ATP-pocket of KIT/PDGFRA only in the inactive formation. Secondary mutations in the activation loop induce a shift towards the active confirmation, reducing the ability of these drugs to bind to the kinase (13). Second, complex intra- and intertumor heterogeneity contribute to drug resistance, making global tumor control difficult. Early GIST TKIs inhibit certain mutations in KIT and PDGFRA, but they do not inhibit all mutations, and in particular, have limited activity against activation loop mutations. Two recently approved next-generation TKIs, avapritinib and ripretinib, were specifically developed to address these issues.

## Next-Generation Novel TKIs Ripretinib and Avapritinib Have Unique MOAs

### Ripretinib (DCC-2618)

**Figure 2:**
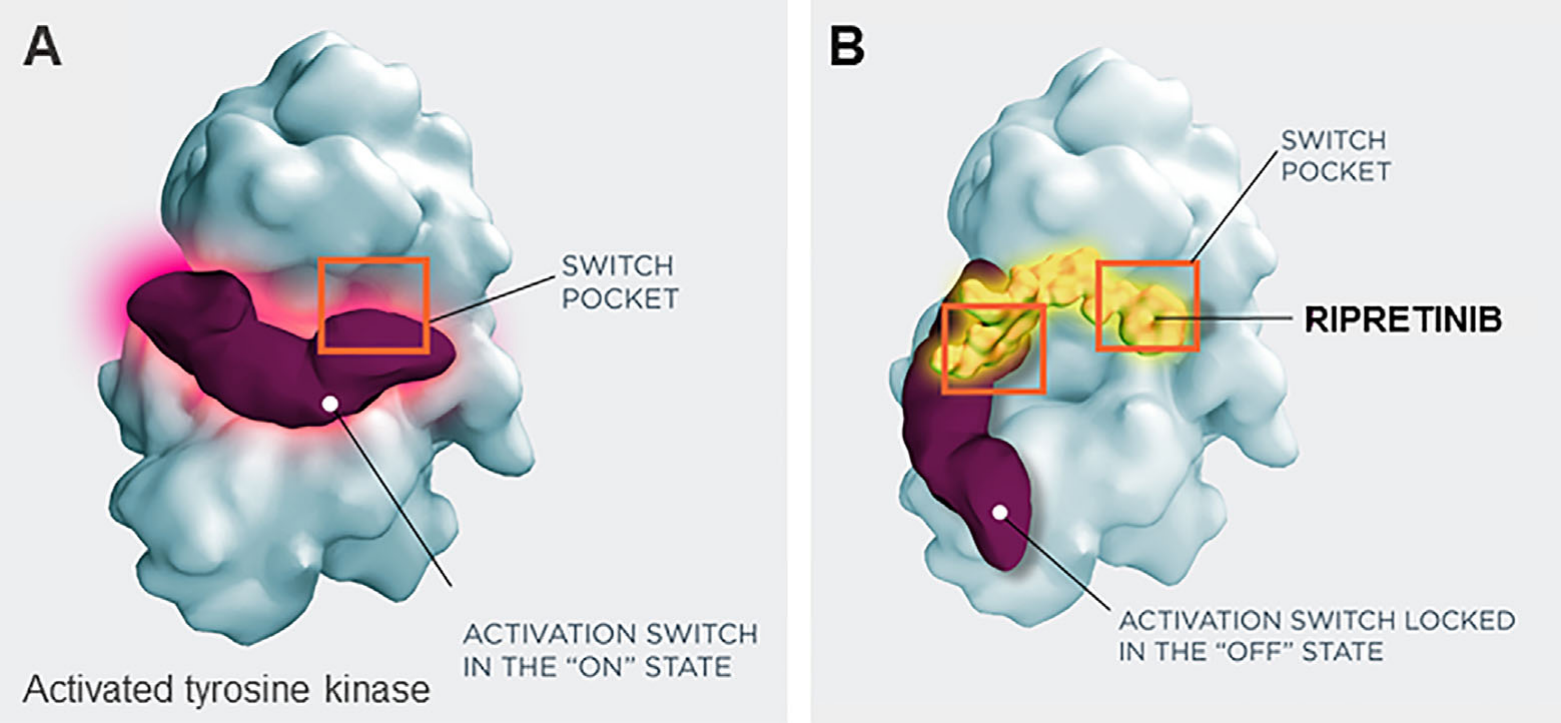
Switch control inhibition by ripretinib. **(A)** Activated tyrosine kinase, **(B)** inactivated tryosine kinase, with ripretinib. Ripretinib uses a dual mechanism of action that secures the kinase into an inactive confirmation and prevents downstream signaling by binding both the switch-pocket region and the activation switch.

Ripretinib received FDA approval on May 15, 2020, for the treatment of adult patients with advanced GIST who have received prior treatment with 3 or more kinase inhibitors, including imatinib (57). Ripretinib is a novel type II switch control kinase inhibitor with a dual MOA, regulating both the kinase switch-pocket and activation loop (**Figure 2**) (14). Ripretinib functions by binding to the switch pocket, preventing access by the activation loop and thus blocking the kinase from adopting an active state. This results in the inhibition of downstream signal transduction (14). Ripretinib was designed as a broad-spectrum KIT and PDGFRA kinase inhibitor that inhibits the full spectrum of primary and secondary drug resistance mutations, including activation loop mutations previously thought to be targeted only by type I inhibitors (14).

Preclinical studies of ripretinib showed significant antitumor activity in GIST and mastocytosis models, including inhibition of proliferation and induction of apoptosis (14, 58). In a preclinical study, ripretinib displayed a broader spectrum of inhibition and, compared with early TKIs, was a potent inhibitor of both wild-type and mutant KIT and PDGFRA kinases, including *PDGFRA* exon 18 D842V mutation (14).

The first human trial, a two-part (dose escalation and dose expansion) phase 1 study, assessed safety and tolerability of ripretinib in advanced GIST with KIT or PDGFRA mutations that had at least one prior line of therapy (59). No maximum tolerated dose was reached during the study, with less than 33% of patients at each dose level experiencing a dose-limiting toxicity (59). The recommended phase 2 dose was established at 150 mg once daily, based on preclinical pharmacology studies predicting 150 mg once daily to be an effective dose, as well as the initial pharmacokinetics analysis. Peak plasma concentration [mean C_max_ (coefficient of variation %)] following a single dose of 150 mg ripretinib on cycle 1 day 1 was determined to be 502 ng/mL (56.8%) and exposure (AUC_0-24h_) was 6634 ng x h/mL (59.8%) (59). Overall, in the phase 1 trial, ripretinib had a favorable safety profile and was generally well tolerated; the most common TEAE was alopecia (62%) (59). In patients on second-line treatment, the mPFS was 10.7 months; as a third-line treatment, the mPFS was 8.3 months; for fourth-line, the mPFS was 5.5 months (59). Among patients receiving 150 mg once daily, upon disease progression, they had the option to dose escalate to 150 mg twice daily. Dose escalation demonstrated an additional PFS clinical benefit across all treatment lines with a safety profile similar to that observed at the once-daily dose (60).

The efficacy of ripretinib was further evaluated in the INVICTUS study (NCT03353753), a double-blind, randomized, placebo-controlled phase 3 trial assessing ripretinib in patients with advanced GIST who had progressed on at least imatinib, sunitinib, and regorafenib (61). As a fourth-line or later treatment, ripretinib significantly increased mPFS over placebo (6.3 months *vs* 1 month, respectively; HR 0.15, 95% CI 0.09–0.25; p <0.0001) (61). The median OS was 15.1 months in the ripretinib group compared to 6.6 months in the placebo group. For patients in the ripretinib arm, 8 (9%) had a partial response, 56 (66%) had stable disease at 6 weeks, and 40 (47%) had stable disease at 12 weeks, compared with patients in the placebo arm, which showed 0 patients with a partial response, 9 (20%) with stable disease at 6 weeks, and 2 (5%) with stable disease at 12 weeks (61). Patients in the INVICTUS study also had the option to dose escalate to 150 mg twice daily. Of the 43 patients in the ripretinib arm that dose escalated, mPFS before dose escalation was 4.6 months and after escalation mPFS was 3.7 months, providing further support for the potential of dose escalation with ripretinib following disease progression (62). A third clinical trial—the randomized, open-label, phase 3 INTRIGUE (NCT03673501) trial—completed accrual in December 2020, comparing the safety and efficacy of ripretinib to sunitinib in patients with advanced GIST who have progressed on imatinib (61, 63). This trial is poised to provide some important information on the efficacy of ripretinib on secondary mutations. In addition, effective salvage therapies may shift based upon the differential activity of ripretinib as compared with sunitinib, with the spectrum and distribution of resistance mutations likely differing between the 2 cohorts after treatment with ripretinib or sunitinib.

While ripretinib shows improved survival in GIST patients, even following treatment with all approved agents, and has a proposed broad-spectrum inhibition for primary and secondary mutations, patients continue to show disease progression. This disease progression may be due to yet unidentified resistance mechanisms; it has been speculated that potential resistance mechanisms in ripretinib could involve ATP-binding pocket resistance mutations (10). In addition, when strong KIT/PDGFRA inhibition is achieved, emergence of KIT-independent resistance mechanisms is likely to occur, potentially involving mutations of PI3K, RAS/RAF, TSC1 and 2, and NF1 (36). Alternatively, not all mutations may be maximally inhibited. A recent study has suggested that ripretinib may only have modest activity against exon 13 and 14 *KIT* mutations in an assay performed in the presence of physiological amounts of human serum albumin and alpha1-acid glycoprotein (64), which may explain why the PFS in the INVICTUS study was not longer. This is in contrast to what was reported in Smith et al. (14), which showed strong inhibition of these mutations. However, there were methodological differences between these two studies, including the amount and type of serum protein in the assay system.

### Avapritinib (BLU285)

Avapritinib, a selective, small-molecule inhibitor of KIT and PDGFRA activation loop mutants, was approved in January 2020 by the FDA for treatment of GISTs that harbor a *PDGFRA* exon 18 mutation, including D842V mutations (65). Notably, it was the first TKI approved with efficacy for GIST with the *PDGFRA* D842V mutation and is more potent (~10 fold) against this mutation than ripretinib (14). Avapritinib is a competitive ATP-binding site inhibitor; however, it was designed to specifically interact with the active conformation (type I kinase inhibitor), unlike the early type II kinase inhibitors that only bind to the inactive conformation (13).

In both *in vitro* and *in vivo* preclinical testing, avapritinib demonstrated potency across a spectrum of primary and secondary mutations, including the difficult-to-treat *PDGFRA* D842V mutation (13, 66). In preclinical murine models with patient-derived GIST xenografts (UZLX-GIST9^KIT11+17^; UZLX-GIST3^KIT11^GIST3^KIT11^; UZLX-GIST2B^KIT9^GIST2B^KIT9^), avapritinib reduced tumor volume and inhibited proliferation (66).

The safety and efficacy of avapritinib was evaluated in 2 clinical trials, NAVIGATOR and VOYAGER. The NAVIGATOR trial was an open-label, 2-part, dose escalation and dose expansion phase 1 trial of patients with advanced GIST (67). Part 1 of the study, dose escalation, included patients with advanced GIST who were refractive to imatinib and at least one other TKI. Part 2, dose expansion, had several cohorts, including one restricted to patients with advanced GIST with the *PDGFRA* D842V mutation, regardless of prior therapy status (67). Part 1 enrolled 46 patients, of whom 20 had *PDGFRA* D842V mutant GIST; part 2 enrolled 36 patients with *PDGFRA* D842V mutant GIST (67). Overall, in the *PDGFRA* D842V mutant GIST population (56 patients), 5 (9%) had a complete response, 44 (79%) had a partial response, and 7 (13%) had stable disease; PFS was 81% at 12 months (67). Updated results of the *PDGFRA* D842V mutant GIST population report an overall response rate of 91% (51/56 patients), a median PFS of 34 months, and the median OS not yet reached (68). In this study, avapritinib had an acceptable safety profile, with AEs typically Grade 1 or 2. Two AEs of special interest were identified: cognitive effects (40%, including memory impairment, cognitive disorder, confusional state, and encephalopathy) and intracranial bleeding (2%) (67). The majority of cognitive effects were Grade 1 and led to discontinuation of treatment in 2 patients (67). For effective management of neurocognitive side effects greater than Grade 1, patients need immediate dose reductions or interruptions, which requires close monitoring, in order to allow long-term treatment in a GIST genotype for which no other treatment is available (65).

Very recently, mechanisms of secondary resistance to avapritinib in PDGFRA-mutant GIST have been described (69). They involve compound mutations of exons 13, 14, and 15 of *PDGFRA* that show cross-resistance to all other drugs that inhibit PDGFRA (69).

The VOYAGER trial (NCT03465722) was an open-label, randomized, phase 3 trial comparing avapritinib and regorafenib in patients with metastatic GIST previously treated with imatinib and 1 or 2 other TKIs (70). A press release from the study sponsor indicated that the VOYAGER trial did not meet its primary endpoint of increased PFS in patients treated with avapritinib compared to regorafenib. The reported PFS with avapritinib was 4.2 months, which was not significantly different from regorafenib at 5.6 months (70).

## Intra- and Intertumoral Heterogeneity and Clonal Evolution Complicates Treatment

Tumor heterogeneity is a major issue in cancer treatment because it contributes to the variable response between patients. As discussed above, exposure to imatinib exerts pressure on a tumor that can trigger the selection and expansion of clones with secondary mutations (71). In patients with GIST primary mutations, tumors harboring multiple secondary kinase mutations are reported in approximately 19% – 44% of patients; up to 70% of patients have GIST with ≥2 different mutations in separate metastases (12, 41, 72, 73). As TKIs have selective efficacy for different mutations, intra- and intertumoral heterogeneity result in mixed responses to treatment. This may explain why PFS is shorter with subsequent TKIs post-imatinib. Resistance to second-line and greater TKIs may result from pre-existing small clones or new mutations driven by selective pressure. Whole genome sequencing in TKI-resistant GIST has revealed a subset of patients who harbor mutations in intermediates of KIT-downstream signaling that are potentially involved with resistance (36).

Heterogeneity can be difficult to detect and is likely underestimated, as samples cannot be taken from every tumor. Currently, tissue biopsy is the gold standard for molecular characterization (74); however, it provides a static picture of one part of the tumor at a single point in time and repeated sampling is not feasible for these patients (74). Liquid biopsy combined with sequencing techniques, such as next-generation sequencing, is an emerging technology that has the potential to address these issues. Circulating tumor-associated molecules within the blood such as circulating tumor DNA (ctDNA) and circulating tumor cells can be captured and sequenced to monitor the evolution of GIST mutations and identify those associated with drug resistance (74). The majority of liquid biopsy studies in GIST have assessed ctDNA (for a detailed review, see Gómez-Peregrina et al.) (75). Patients with localized disease were less likely to have detectable ctDNA, whereas those with high-burden metastatic disease that was progressing showed the highest rate of ctDNA detection (76). Overall, it is difficult to make direct comparisons between studies, as they have different methodologies. The data suggests, however, that there is potential for the utility of liquid biopsy in GIST, and there is ongoing research to optimize this utility in this patient population (74, 75). In a recent study, next-generation sequencing of liquid biopsies of GIST from treated patients with high tumor burden confirmed that multiple resistance mutations can be simultaneously present and detected in ctDNA shed from tumors (77). Secondary mutations were diverse and determined to be spatially distributed in the tumors *via* detailed analysis of tissue from surgical resection (77). For one patient in the study, comprehensive analysis of repeated plasma samples over 53 weeks identified intratumor heterogeneity and polyclonal evolution that would not have been captured with tissue biopsy (77). In the INVICTUS trial, combining tumor and liquid biopsy increased the rate of detection for resistance mutations, allowing detection in 73% of patients (78). Plasma sequencing from these liquid biopsies revealed secondary resistance mutations in at least 1 exon in 70% of KIT/PDGFRA primary mutant GIST, and 24% of patients had 2 or more exons affected, with up to 4 exon mutations detected in 1 patient (78). These results highlight the heterogeneity of GIST tumors. Liquid biopsy has also been correlated with treatment response. The NAVIGATOR trial, which assessed avapritinib in patients with GIST, showed lower baseline ctDNA levels were associated with prolonged PFS (79). However, despite its potential, liquid biopsy is in the early stages of development for GIST and is not yet validated as a tool for clinical decision-making. Given the role of mutations in GIST, it is likely that the combined results from primary tumor genotyping, ctDNA, and tumor biopsies will be used in the future to guide treatment decisions for patients with TKI-resistant disease. Therefore, patients would benefit from treatment with a multidisciplinary team that routinely evaluates and uses such data in their clinical decision making.

## Summary of Advanced GIST Treatments and Future Steps

KIT/PDGFRA inhibition remains the backbone of therapy for metastatic GIST due to the underlying oncogenic drivers of this disease. Despite providing significant clinical benefit, resistance and disease progression limit patient survival, with secondary KIT mutations as the major driver of resistance. In 2020, two new TKIs, avapritinib and ripretinib, were approved for advanced GIST (57, 65). Both have unique MOAs and were designed to address known mechanisms of resistance to early TKIs. Ripretinib, as a switch-pocket inhibitor may inhibit a broader range of mutations, while avapritinib, a type 1 kinase inhibitor, clearly provides benefit for the previously treatment-resistant PDGFRA D842V mutation. **Table 1** presents a summary of the key differences between the available treatments.

**Table 1:** Summary of approved treatments for advanced GIST.

	Imatinib	Sunitinib	Regorafenib	Ripretinib	Avapritinib
Indication for advanced GIST	1st line	2nd line	3rd line	4th line	PDGFRA Exon 18 mutant (including D842V)
MOA	Type II^a^	Type II^a^	Type II^a^	Type II^a^	Type I^b^
	Competitive ATP-binding site inhibitor	Competitive ATP-binding site inhibitor	Competitive ATP-binding site inhibitor	Switch-pocket inhibitor	Competitive ATP-binding site inhibitor
Efficacy					
mPFS (mo)	18	5.6	4.8	6.3	34 ^c^
ORR (%)	50	7	4.5	9.4	91^c^

a Binds the inactive confirmation.

b Binds the active confirmation.

c D842V patients only.

GIST, gastrointestinal stromal tumor; mo, months; MOA, mechanism of action; mPFS, median progression-free survival; ORR, overall response rate; PDGFRA, platelet-derived growth factor receptor a.

### Future of Advanced GIST Treatment

A major challenge in GIST treatment is polyclonal resistance, whereby a single drug is insufficient to target all resistant KIT mutations, thus resulting in tumor progression. While ripretinib has a broad spectrum of inhibition compared to early TKIs, disease progression still occurs. In an attempt to address the need for multiple TKIs to cover the heterogeneity of the tumors, clinical trials have investigated TKI treatments with rapid alternation for tumors with polyclonal secondary mutations. In the SURE study (NCT02164240), a phase 1/2, open-label trial, rapid alternation of sunitinib and regorafenib was used to treat advanced GIST that was refractory to imatinib (80). Sunitinib and regorafenib were chosen because of their complementary inhibition profiles for KIT mutations (80). Patients experienced 3 days of sunitinib followed by 4 days of regorafenib (80). Of 13 enrolled patients, 4 had stable disease, and the mPFS for all patients was 1.9 months, which is similar to rechallenge with imatinib (80). All 13 patients experienced treatment-related AEs, the majority of which were Grade 1 or 2. The most common AEs were fatigue (92%), weight loss (62%), hand-foot syndrome (54%), anorexia (38%), hypertension (38%), and hoarseness (38%) (80). Rapid alteration with other TKIs may represent a viable strategy in the future for the treatment of advanced GIST. The possibility of using the new generation TKIs with more favorable safety profiles, like ripretinib, in such a scenario is an appealing possibility. Another combination of 2 drugs with complementary activity that showed promise is PLX9486 (a selective TKI now known as CGT9486) with sunitinib, yielding a mPFS of 12 months in the initial study report (81). Other future strategies to overcome polyclonal resistance using currently approved drugs may include combination treatment. For example, preliminary results of the combination of imatinib and binimetinib for first-line treatment of advanced GIST has shown promise; of 38 patients, 26 had a partial response (82). Additional future strategies may include more complex scheduling or brief periods of using a triple combination of anti-cancer therapies.

Additionally, other targets are in development, including heat-shock protein 90 (HSP90) inhibitors. HSP90 inhibitors can induce KIT degradation and thus represent a potential therapy for GIST (83). Early HSP90 inhibitors had issues with hepatotoxicity and neurological toxicities (83). However, newer HSP90 inhibitors such as TAS-116 are showing promise in both preclinical and clinical trials for antitumor activity and more manageable AEs with intermittent dosing (83–85). Finally, reports showing adaptive and innate immune cells in the GIST tumor microenvironment also suggest immunotherapy may be a potential future treatment for GIST (86). Anti-human CD117 chimeric antigen receptor T-cells were recently shown to eliminate healthy and malignant CD117-expressing hematopoietic cells (87); these cells may have potential for malignant CD117-expressing cells in GIST as well, but more research is needed.

In conclusion, KIT and PDGFRA, as the oncogenic drivers of GIST, are still viable targets for therapy as demonstrated by the approval of ripretinib and avapritinib. Their unique MOAs have helped address some of the limitations of early TKI treatments. Despite improved outcomes, polyclonal resistance and tumor heterogeneity still lead to disease progression, and continued research to overcome these challenges is necessary.

## Author Contributions

SB and MCH devised the main conceptual ideas and outline. All authors contributed to the article and approved the submitted version.

## Funding

The authors received no financial support for the research, authorship, and/or publication of this article. Funding for editorial support was provided by Deciphera Pharmaceuticals, LLC. Medical writing and editorial support were provided by Helen Rodgers, PhD, of AlphaBioCom, LLC, King of Prussia, PA, USA, and funded by Deciphera Pharmaceuticals, LLC, Waltham, MA, USA. MCH received partial salary support from Veterans Affairs Merit Review Grants (2I01BX000338-05 and 1I01BX005358).

## Conflict of Interest

SB has received honoraria from Bayer, Eli Lilly, Novartis, Pfizer, and PharmaMar, serves in an advisory/consultancy role for ADC Therapeutics, Bayer, Blueprint Medicines, Daiichi Sankyo, Deciphera Pharmaceuticals, Eli Lilly, Exelixis, Janssen-Cilag, Nanobiotix, Novartis, PharmaMar, Plexxikon, and Roche, receives research funding from Novartis, and serves as a member of the External Advisory Board of the Federal Ministry of Health for “Off-label use in oncology.” SG serves in an advisory/consultancy role for AstraZeneca, Bayer, Blueprint Medicines, Daiichi Sankyo, Deciphera Pharmaceuticals, Eli Lilly, and Exelixis, has a leadership role in Alliance Foundation, receives licensing royalties from Wolters Kluwer Health, is a shareholder/stockholder of Abbott Labs and Allergan, and her institution receives research support from Bayer, Blueprint Medicines, Deciphera Pharmaceuticals, Novartis, and Pfizer. MVM serves in an advisory/consultancy role for Deciphera Pharmaceuticals, Blueprint, Exelexis, has received travel/accommodation expenses from Deciphera Pharmaceuticals, and NCCN and her institution has received funding from Arog, ASCO, Blueprint, Deciphera Pharmaceuticals, Garadalis, GenMab, Novartis, and Solarius. MCH serves in a consultancy role for Blueprint, Deciphera Pharmaceuticals, and Novartis, receives royalties from Novartis, receives grant funding from Blueprint, and Deciphera Pharmaceuticals, and has received travel, accommodations, expenses from Blueprint, and Deciphera Pharmaceuticals.

The reviewer CS declared a past co-authorship with the authors to the handling Editor.

## Abbreviations

GIST, gastrointestinal stromal tumor; PDGFRA, platelet-derived growth factor receptor α; RTK, receptor tyrosine kinase; VEGFR, vascular endothelial growth factor receptor.
